# Survival status of women with cervical cancer in Sub-Saharan Africa: a systematic review and meta-analysis, 2024

**DOI:** 10.3389/fonc.2024.1491840

**Published:** 2025-01-07

**Authors:** Tadele Emagneneh, Chalie Mulugeta, Betelhem Ejigu, Abebaw Alamrew, Aynalem Yetwale Hiwot, Sefineh Fenta Feleke

**Affiliations:** ^1^ Department of Midwifery, College of Health Sciences, Woldia University, Woldia, Ethiopia; ^2^ Department of Public Health, College of Health Sciences, Woldia University, Woldia, Ethiopia

**Keywords:** cervical cancer, survival status, Sub-Saharan Africa, systematic review, meta-analysis

## Abstract

**Background:**

Despite the availability of vaccination and early treatment, cervical cancer remains a significant public health concern globally, particularly in Sub-Saharan Africa, where access to screening and treatment is often limited.

**Methods:**

In this study, researchers conducted a survey of four international databases—Medline/PubMed, Scopus, Web of Science, and Henare—along with Google Scholar to search for gray literature. The keywords used for searching the international databases included “Uterine Cervical Neoplasms [Mesh],” “Survival OR Survival Analysis OR Survival Rate,” and “Sub-Saharan countries” (including the names of specific countries). Six researchers independently screened and extracted data from the articles. All studies published in English were included without restriction and assessed for quality using the adapted Newcastle–Ottawa Scale for cohort and cross-sectional studies. The results of this systematic review were reported in accordance with the PRISMA checklist.

**Results:**

Out of the 2,180 articles initially identified, 23 were deemed eligible and reported on the survival status of patients with cervical cancer in Sub-Saharan Africa. This study assessed the multi-year survival rates (1, 2, 3, 4, and 5 years) of patients with cervical cancer. Based on the random-effects model, the overall pooled 1-year survival was 65.0% [95% confidence interval (CI), 52–78] with I² = 99.31 and p-value < 0.001. The 2-year survival rate was 60% (95% CI, 46–74) with I² = 99.12 and p-value < 0.001, the 3-year survival was 48% (95% CI, 35–62) with I² = 98.45 and p-value < 0.001, the 4-year survival was 42.9% (95% CI, 32.7–53.1) with I² = 96.80 and p-value < 0.001, and the 5-year survival was 35% (95% CI, 27–44) with I² = 98.74 and p-value < 0.001.

**Conclusions:**

This systematic review and meta-analysis found that the survival rates for patients with cervical cancer in Sub-Saharan Africa are much lower than the global averages. The results show that the 5-year survival rate can be as low as 35%, highlighting serious challenges in managing cervical cancer in this region. To address this issue, collaboration among governments, healthcare providers, and international organizations is essential to enhance the availability and quality of care. Future research should focus on developing effective early detection and treatment strategies and monitoring long-term survival outcomes.

## Introduction

Cervical cancer develops in the cervix, the lower part of the uterus that connects to the vagina, and is caused by the abnormal growth of cells due to infection with certain strains of the human papillomavirus (HPV) (1). Globally, the majority of cervical cancer and pre-cancerous cervical lesions are primarily caused by two specific types of HPV, 16 and 18, which are typically transmitted through sexual contact (2). Early detection of cervical cancer is achievable through regular screening tests like Pap smears, which can significantly increase the chances of successful treatment if the cancer is identified in its early stages (3). Common symptoms include vaginal bleeding (especially after intercourse), abnormal vaginal discharge, and pelvic pain (1). Early treatment options typically involve surgery, radiation therapy, chemotherapy, or a combination of these methods, which can help prevent cervical cancer–related mortality (4).

Despite the availability of vaccination and early treatment, cervical cancer remains a significant public health concern worldwide, particularly in Sub-Saharan countries where access to screening and treatment is often limited (5). It is the fourth most common cancer among women globally, with nearly 12% of all female cancer cases occurring in these regions, accounting for approximately 85% of the global burden (6). In contrast, less than 1% of cervical cancer cases are found in high-income areas (7). Most cases occur in women aged 30 to 50, and cervical cancer is one of the few cancers that can be almost entirely prevented and treated if diagnosed early (3, 4).

Cervical cancer is the second most common cancer among women globally and is the leading cause of cancer-related deaths in developing countries (5). It ranks as the third most common cancer in women, following breast and colorectal cancers, and is a major cause of cancer death worldwide (8). In 2018, it was the fourth most common cancer, representing about 6% of all female cancer cases (9). Additionally, cervical cancer was the fourth leading cause of cancer deaths, accounting for 8% of all female cancer deaths globally (10). The disease contributes to an estimated 9.0 million disability-adjusted life years, indicating a significant public health burden (11, 12). Cervical cancer remains a pressing global health issue, with a particularly severe impact in many low- and middle-income countries (13).

In many developed countries, cervical cancer is gradually becoming a rare disease, but this is not the case in many Sub-Saharan African countries (11). In Sub-Saharan Africa, cervical cancer is the most common cancer among women and ranks second only to breast cancer in northern Africa (5). It constitutes 22.2% of all cancers in women in the region and remains the leading cause of cancer-related deaths among women (14). Approximately 60%–75% of women with cervical cancer in Sub-Saharan Africa live in rural areas, where the mortality rate is extremely high (11).

A significant number of women with cervical cancer in Sub-Saharan Africa do not receive treatment, primarily due to barriers in accessing healthcare, both financial and geographical (14). Women in the region lose more years of life to cervical cancer compared to any other cancer (2). Unfortunately, it affects them at a time of life when they are critical to the social and economic stability of their families (13, 14). The incidence of cervical cancer remains alarmingly high in Sub-Saharan Africa, with rates up to 15 times higher in low-income countries compared to that in industrialized nations (15). In the year 2000, there were an estimated 57,000 cases of cervical cancer, which represented 22.2% of all cancers in women, translating to an age-standardized incidence rate of 31 per 100,000 (8).

Mortality rates from cervical cancer in Africa are also very high, with Eastern Africa reporting a rate of 35 per 100,000 (16). In 1990, the 5-year relative survival rates for cervical cancer were 18% in Kampala, Uganda, and 30% in Harare, Zimbabwe, compared to 72% in the USA during the same period (16). In Harare, 77% of 284 registered patients with cervical cancer died within 3 years of follow-up (17). The 3-year overall observed and relative survival rates were 44.2% and 45.2%, respectively (17). By 2002, the survival rate for cervical cancer in Sub-Saharan Africa was 21%, whereas it was 70% in the United States.

The high mortality and low survival rates for cervical cancer in Sub-Saharan Africa are attributed to various factors, including limited access to medical facilities, particularly in rural areas where 60%–70% of affected women reside. Other contributing factors include poor nutrition and co-morbid conditions like anemia and malaria, HIV infection, late-stage disease presentation, large tumor size at diagnosis, substandard quality of care in many health services, high rates of loss to follow-up, and treatment non-completion due to poverty-related barriers (2, 11, 13, 18, 19).

Research into cervical cancer survival across different cultural, racial, and genetic populations has demonstrated that these factors influence multi-year survival status of patients with cervical cancer. As there has been no comprehensive study assessing the multi-year survival rate of patients with cervical cancer in Sub-Saharan Africa, this study aims to conduct a systematic review and meta-analysis to determine multi-year survival rates in the region. This information is intended to aid in the development of effective public health interventions for the prevention, diagnosis, and treatment of cervical cancer.

## Methods

This study is a systematic review and meta-analysis focused on cervical cancer survival rates in Sub-Saharan countries. Conducted in 2024, the study follows the PRISMA (Preferred Reporting Items for Systematic Reviews and Meta-Analyses) guidelines for reporting (2).

### Search strategy/methodology

In this study, researchers conducted a survey of four international databases—Medline/PubMed, Scopus, Web of Science, and Henare—along with Google Scholar, to identify articles published by 15 August 2024. Google Scholar was also used to search for gray literature. The keywords used for searching the international databases included “Uterine Cervical Neoplasms [Mesh],” “Survival OR Survival Analysis OR Survival Rate,” and “Sub-Saharan countries” (names of countries) (see **Supplementary Table 1**). The data collected were entered into Mendeley software, which automatically removed duplicate articles. Six researchers independently reviewed the articles. The search strategy is detailed in **Supplementary Table 1**.

### Inclusion and exclusion criteria

The study included all observational studies (cross-sectional, case-control, and cohort) published up to 15 August 2024 that reported on the survival of cervical cancer and were published in English, with no time limit applied. Review studies and meta-analyses were excluded from the selection. Additionally, studies that did not provide sample sizes or did not report confidence intervals (CIs) for survival of cervical cancer were excluded from the meta-analysis.

### Screening of studies

Initially, the articles retrieved from the selected databases were imported into the Mendeley library, where exact duplicates were removed. The Mendeley library was then shared among six authors, who independently screened the articles by title and abstract. Following the abstract review, Cohen’s kappa coefficient was calculated to assess the level of agreement among the reviewers. A substantial agreement was deemed acceptable if the Cohen’s kappa coefficient was greater than 0.60 (20) from the Cohen’s kappa coefficient obtained. Disagreements among the reviewers were resolved through discussion. After reaching a consensus, the six reviewers independently conducted the full-text review.

### Data extraction form

Six reviewers independently extracted data from the full texts of the selected articles using an adapted Johanna Briggs Institute data abstraction format (21). Data from the final articles included in the study were extracted using a pre-designed checklist. This checklist recorded information such as the authors’ names, publication year, study duration, sample size, study country, and survival rates at 1, 2, 3, 4, and 5 years.

### Quality assessment (evaluation)

The quality of the articles was assessed using the Newcastle–Ottawa Quality Assessment Scale (22), which comprises three sections: (1) Selection (four questions), (2) Comparison (one question), and (3) Result (three questions). Based on the overall score, the articles were classified into three quality categories: Good (three or four stars in the Selection section, one or two stars in the Comparison section, and two or three stars in the Result section); Average (two stars in the Selection section, one or two stars in the Comparison section, and two or three stars in the Result section); and Poor (zero or one star in the Selection section, zero stars in the Comparison section, and zero or one star in the Result section). Only articles that scored seven or more stars, indicating good quality, were included in the final review and analysis (**Table 1**).

**Table 1:** The study characteristics included in this systematic review and meta-analysis.

Authors and years	Countries	Study design	Participants (N)	Survival rates of patients with cervical cancer					Quality
				1 year	2 years	3 years	4 years	5 years	
MacDuffie et al., 2021	Botswana	Prospective cohort	143	**–**	**–**	**–**	–	56.80%	8
S. Grover et al., 2022	Botswana	Prospective cohort	1,043	–	67.2%	–	–	56.40%	8
Aguade et al., 2023	Ethiopia	Retrospective cohort	322	–	–	–	–	63.40%	8
Seifu et al., 2022	Ethiopia	Retrospective cohort	368				31.00%		7
Gashu et al., 2023	Ethiopia	Retrospective cohort	322	–	–	–	30.20%	63.40%	8
J. Kantelhardt et al., 2014	Ethiopia	Prospective cohort	1,059	90.40%	73.60%			44.29%	8
Mebratie et al., 2022	Ethiopia	Retrospective cohort	422				56.20%	14.00%	8
Wassie et al., 2019	Ethiopia	Retrospective cohort	634	92.11%	75.29%	52.92%	38.62%	38.62%	9
Teshome et al., 2024	Ethiopia	Prospective cohort	180	77.00%	42.00%				7
Sifer et al., 2024	Ethiopia	Retrospective cohort	252	96.99%	92.7%	85.90%	68.00%	18.27%	9
E. Gurmu et al., 2018	Ethiopia	Prospective cohort	907					38.48%	8
J. Daniels et al., 2024	Ghana	Retrospective cohort	105	76.50%		51.50%	32.40%	32.40%	7
Y. Nartey et al., 2017	Ghana	Prospective cohort	821	62%		39%		30.00%	7
F. Bertrand et al., 2016	Ghana	Retrospective cohort	923		50%			40%	
D. Osok et al., 2018	Keny	Retrospective cohort	481				35.00%	7%	
E. Mwaliko et al., 2023	Kenya	Retrospective cohort	162	58.0%	49%	45.00%	54.90%		
O. Maranga et al., 2013	Kenya	Prospective cohort	355	58.90%		57.90%			
W. Kiptoo et al., 2013	Kenya	Retrospective cohort	175					17.70%	8
O. Ola et al., 2023	Nigeria	Retrospective cohort	343				28.20%	19.40%	8
Musaet al., 2016	Nigeria	Retrospective cohort	72	32.90%	30.38				7
P. Boniet al., 2023	Côte d’Ivoire	Prospective cohort	353	38.45%			30.90%		
J. DeBoer et al., 2022	Rwanda	Retrospective cohort	379	39.00%		23%			8
A. Elgoraish et al., 2022	Sudan	retrospective cross-sectional	239	48.56				30%	8

### Statistical analysis

Heterogeneity among the studies was assessed using the Cochran test (with a significance level of less than 0.05) in conjunction with the I² statistic. When heterogeneity was present, the random- effects model with the inverse variance method was applied; otherwise, the fixed- effects model was used. If heterogeneity was detected, then a subgroup analysis was conducted on the basis of the countries where the studies were performed and the sample sizes. All analyses were carried out using STATA version 17.

### Outcome measures and data synthesis

The primary outcomes of this study were multi-year survival status of patients with cervical cancer in Sub-Saharan Africa. Subsequently, the reported multi-year survival rates of cervical cancer were categorized into 1-, 2-, 3-, 4-, and 5-year survival rates. The survival status of the patients with cervical cancer reported in various studies was aggregated by pooling the data from the included articles. To account for the true effects across the studies, a random- effects meta-analysis model was used. This model presented the pooled survival status of patients with cervical cancer in 1-, 2-, 3-, 4-, and 5- year survival status of patients with cervical cancer independently.

### Heterogeneity

The I² statistic results indicated significant heterogeneity among the studies. In the analysis of cervical cancer survival status in Sub-Saharan countries, the observed heterogeneity was as follows: for 1-year survival rate, I² was 99.31 with p-value < 0.001; for 2-year survival rates, I² was 98.98 with p-value < 0.001; for 3 -year survival rates, I² was 0 with p-value = 0.0085; for 4-year survival rates, I² was 96.24 with p-value < 0.001; and for 5-year survival rates, I² was 98.72 with p-value < 0.001. A random-effects model was applied to all analyses of the multi-year survival status of patients with cervical cancer (1-, 2-, 3-, 4-, and 5-year survival rates).

## Results

### Study selection

**Figure 1:**
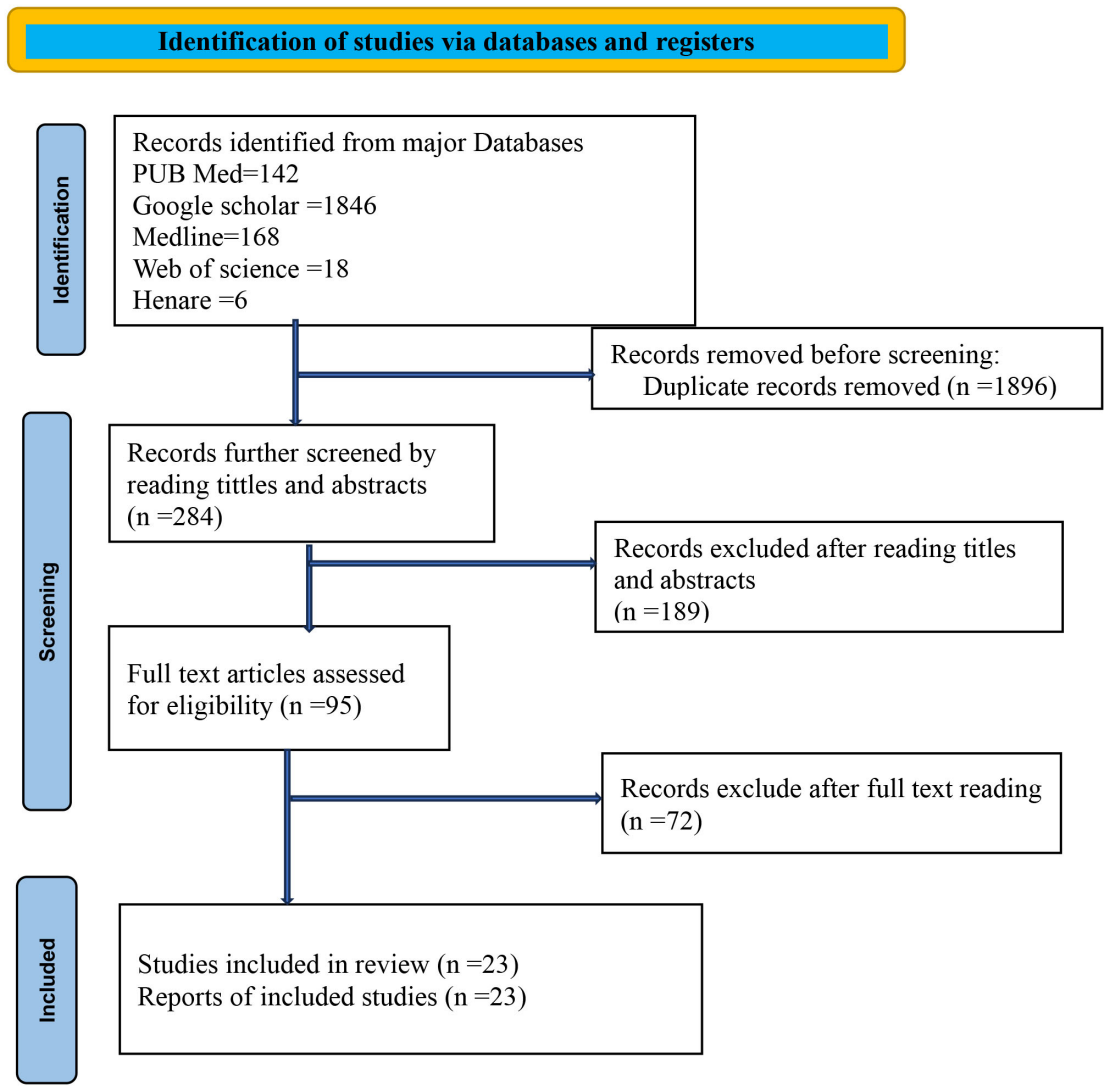
PRISMA flowchart diagram showing the included searches for systematic review and meta-analysis entitled with survival status of cervical cancer in Sub-Saharan countries, 2024.

A total of 2,180 articles were initially identified. After eliminating duplicates, 284 articles were screened on the basis of their titles and abstracts. Following a detailed review, 189 articles were shortlisted for the next stage, where 95 full-text articles were assessed. Ultimately, 23 articles were included in the final analysis. Furthermore, the references of the selected articles were examined to identify additional relevant studies. The process of study selection is illustrated in **Figure 1**.

### Study characteristics

The included studies in the analysis were published up until August 2024, with no limitations on the years of publication. Out of the 2,180 initially identified articles, 23 were deemed eligible and examined for their reports on the survival status of patients with cervical cancer in Sub-Saharan Africa. In terms of study designs, all were cohort studies, except for one cross-sectional study. The distribution of the studies is as follows: nine from Ethiopia, two from Botswana, three from Ghana, four from Kenya, two from Nigeria, one from Côte d’Ivoire, one from Rwanda, and one from Sudan. Further details can be found in **Table 1**.

### Survival rate of patients with cervical cancer in Sub-Saharan Africa

#### One-year survival rate of patients with cervical cancer in Sub-Saharan Africa

**Figure 2:**
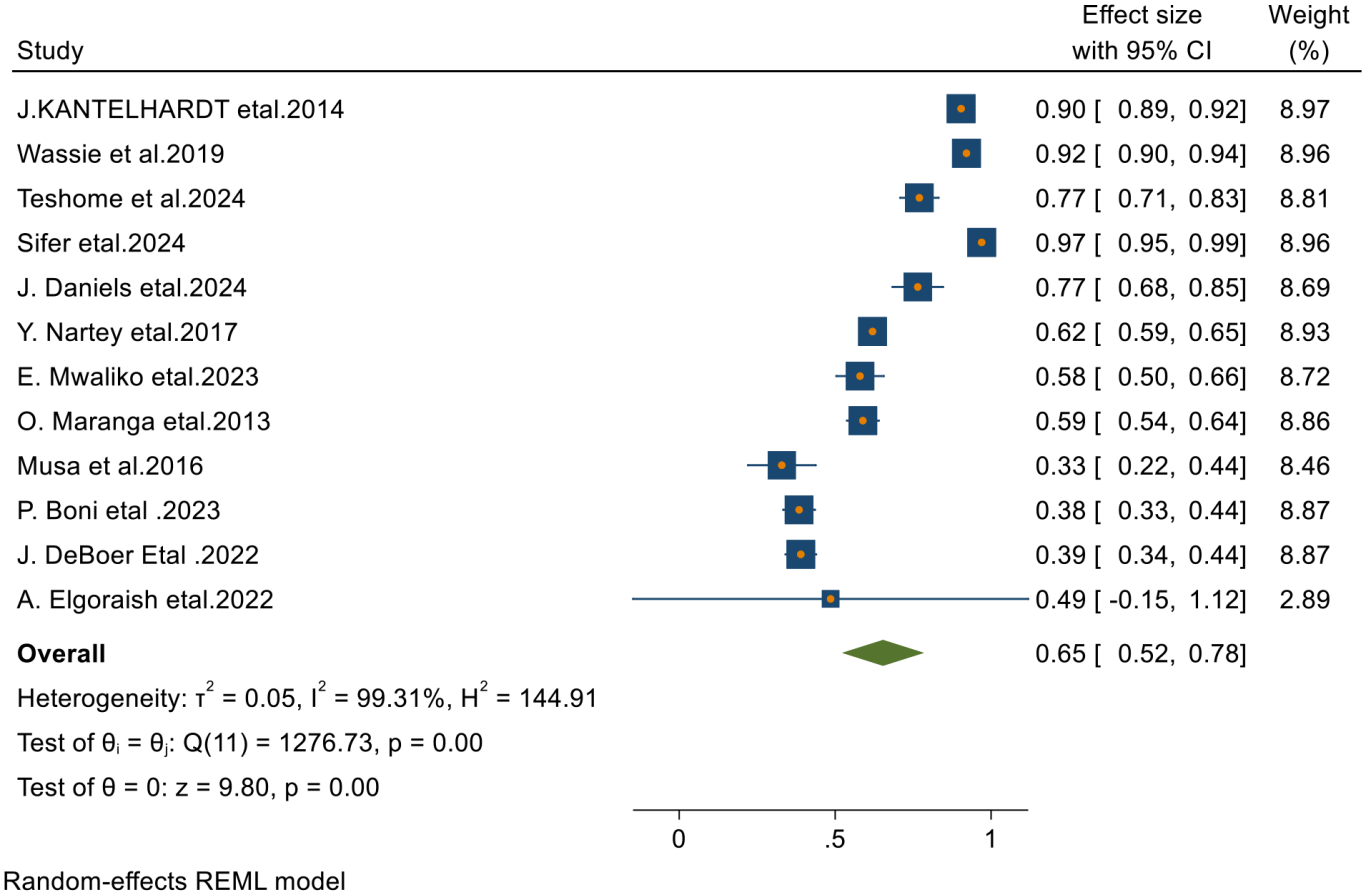
Forest plots of 1-year survival rates of patients with cervical cancer in Sub-Saharan countries.

Of the 23 articles included in the final analysis, 12 studies reported on the 1-year survival rates of patients with cervical cancer. Applying a random-effects model, the overall pooled 1-year survival rate was estimated at 65.0% (95% CI, 52–78), with an I² value of 99.31 and a p-value of less than 0.001 (**Figure 2**).

#### Two-year survival status of patients with cervical cancer in Sub-Saharan Africa

**Figure 3:**
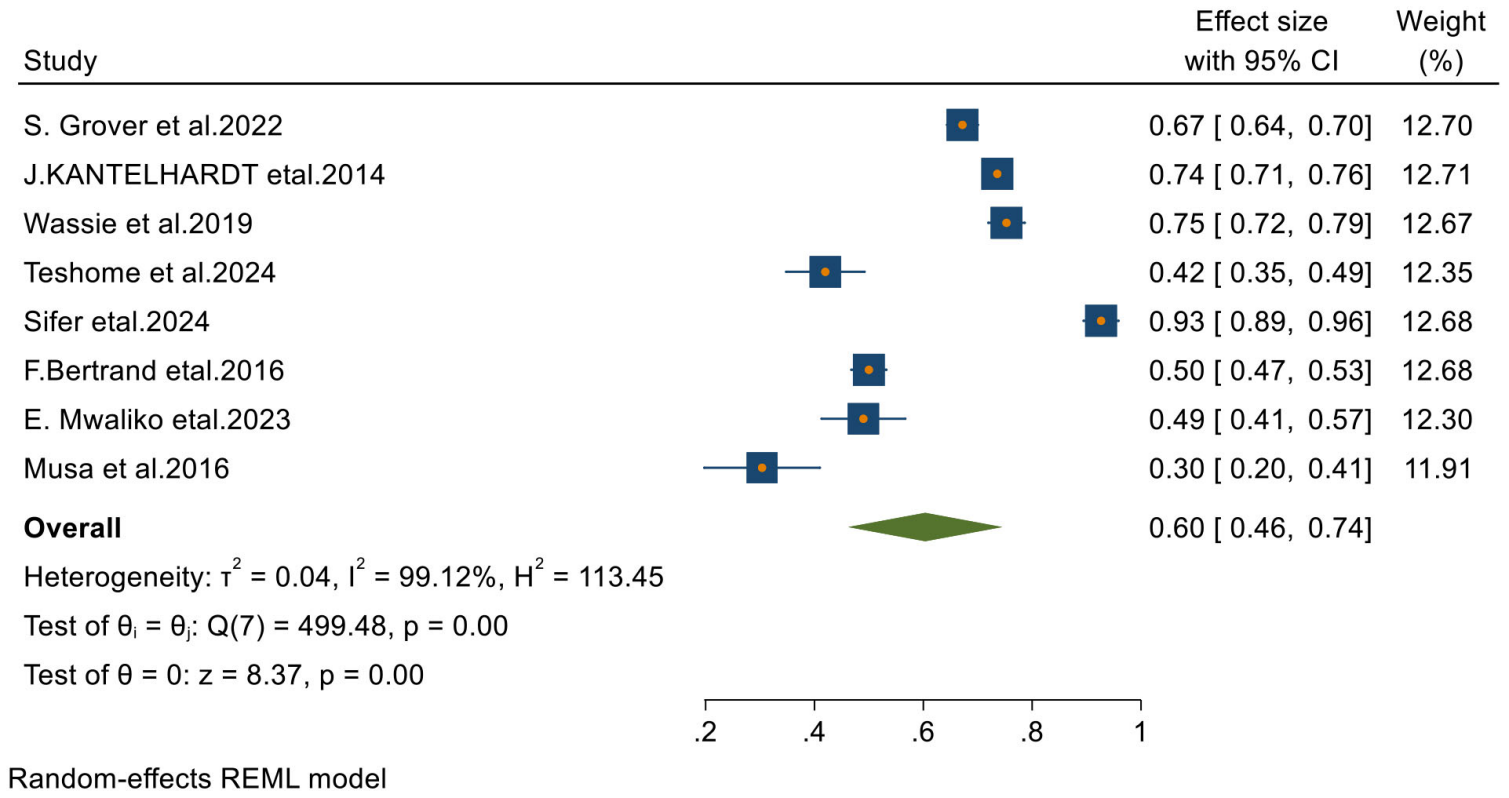
Forest plots of 2-year survival rates of patients with cervical cancer in Sub-Saharan countries.

From 23 final articles that were included in this systematic review and meta-analysis, only 8 studies reported 2-year survival rate of patients with cervical cancer. Based on the random- effects model, the overall pooled 2- year survival status was 60% (95% CI, 46–74) with I^2^ = 99.12 and p-value < 0.001 (**Figure 3**).

#### Three-year survival rate of cervical cancer in Sub-Saharan Africa

**Figure 4:**
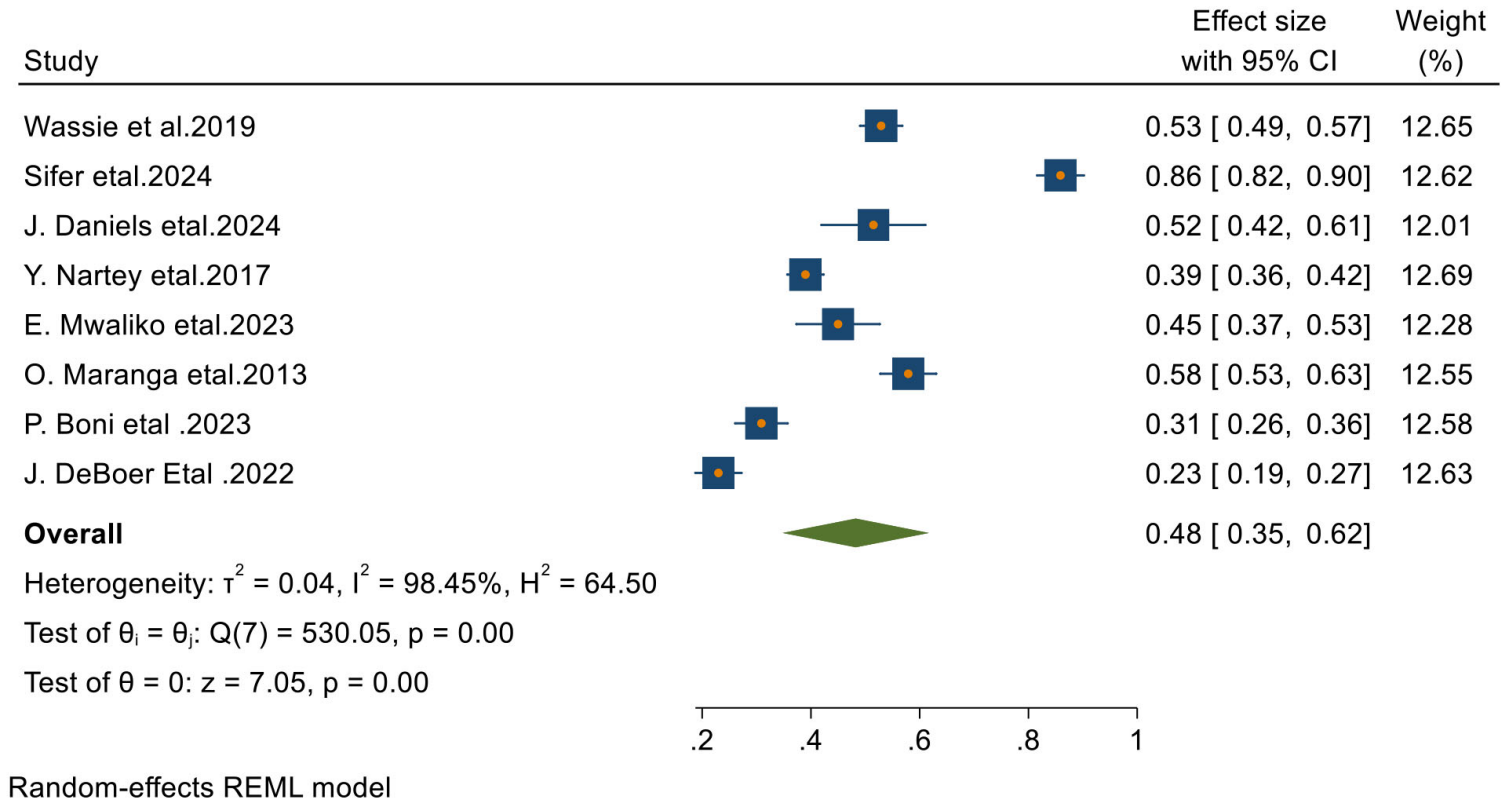
Forest plots of 3-year survival rates of patients with cervical cancer in Sub-Saharan countries.

From 23 final articles that were included in this systematic review and meta-analysis, 8 studies reported 3-year survival rate of patients with cervical cancer. Based on the random- effects model, the overall pooled 3- year survival rate was 48% (95% CI, 35–62) with I^2^ = 98.45 and p-value < 0.001 (**Figure 4**).

#### Four- year survival status of patients with cervical cancer in Sub-Saharan Africa

**Figure 5:**
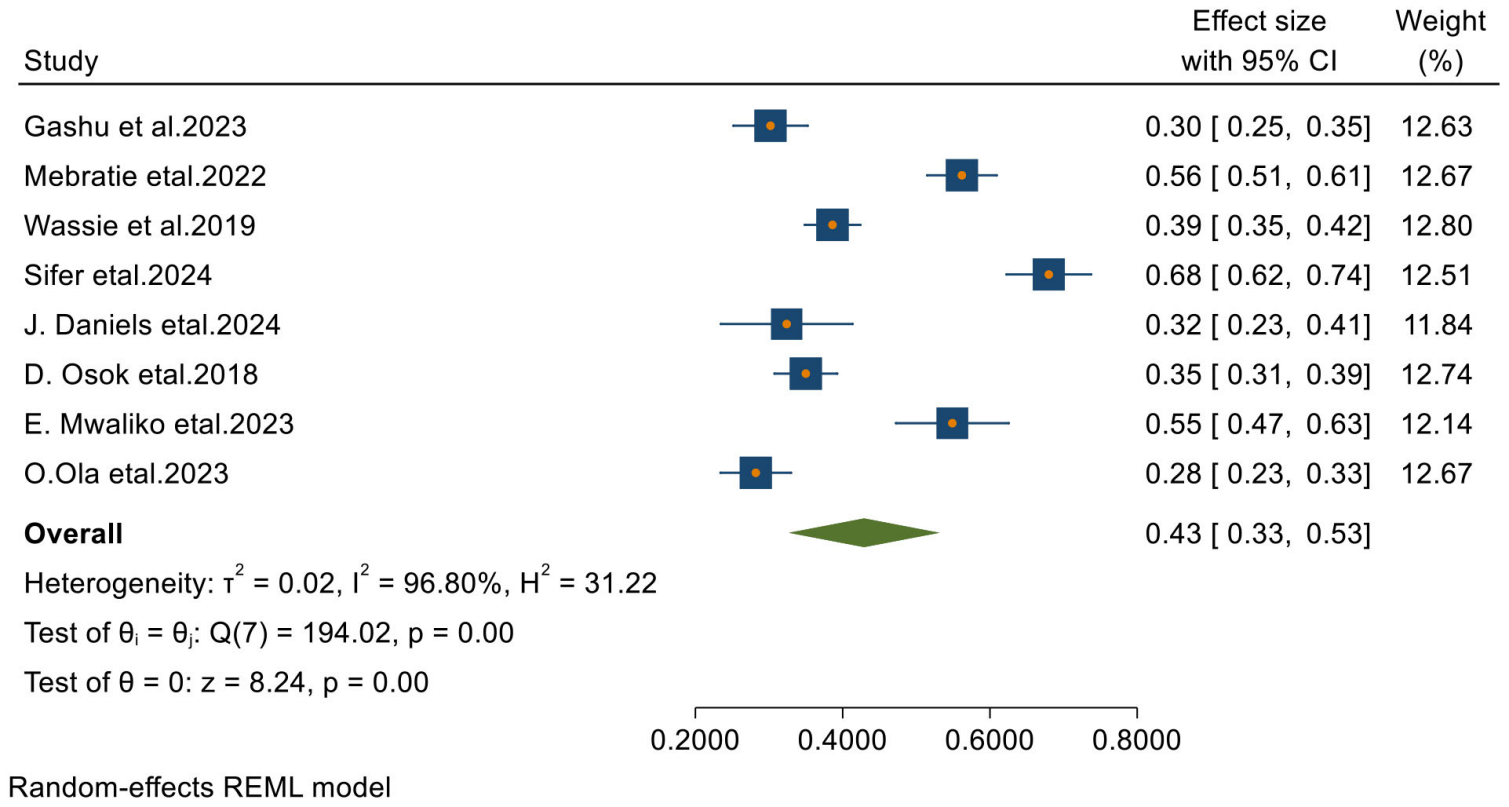
Forest plots of 4-year survival rates of patients with cervical cancer in Sub-Saharan countries.

From 23 final articles that were included in this systematic review and meta-analysis, only 8 studies reported 4-year survival status of patients with cervical cancer. Based on the random- effects model, the overall pooled 4-year survival was 42.9% (95% CI, 32.7– 53.1) with I^2^ = 96.80 and p-value < 0.001 (**Figure 5**).

#### Five- year survival rate of cervical cancer in Sub-Saharan countries

**Figure 6:**
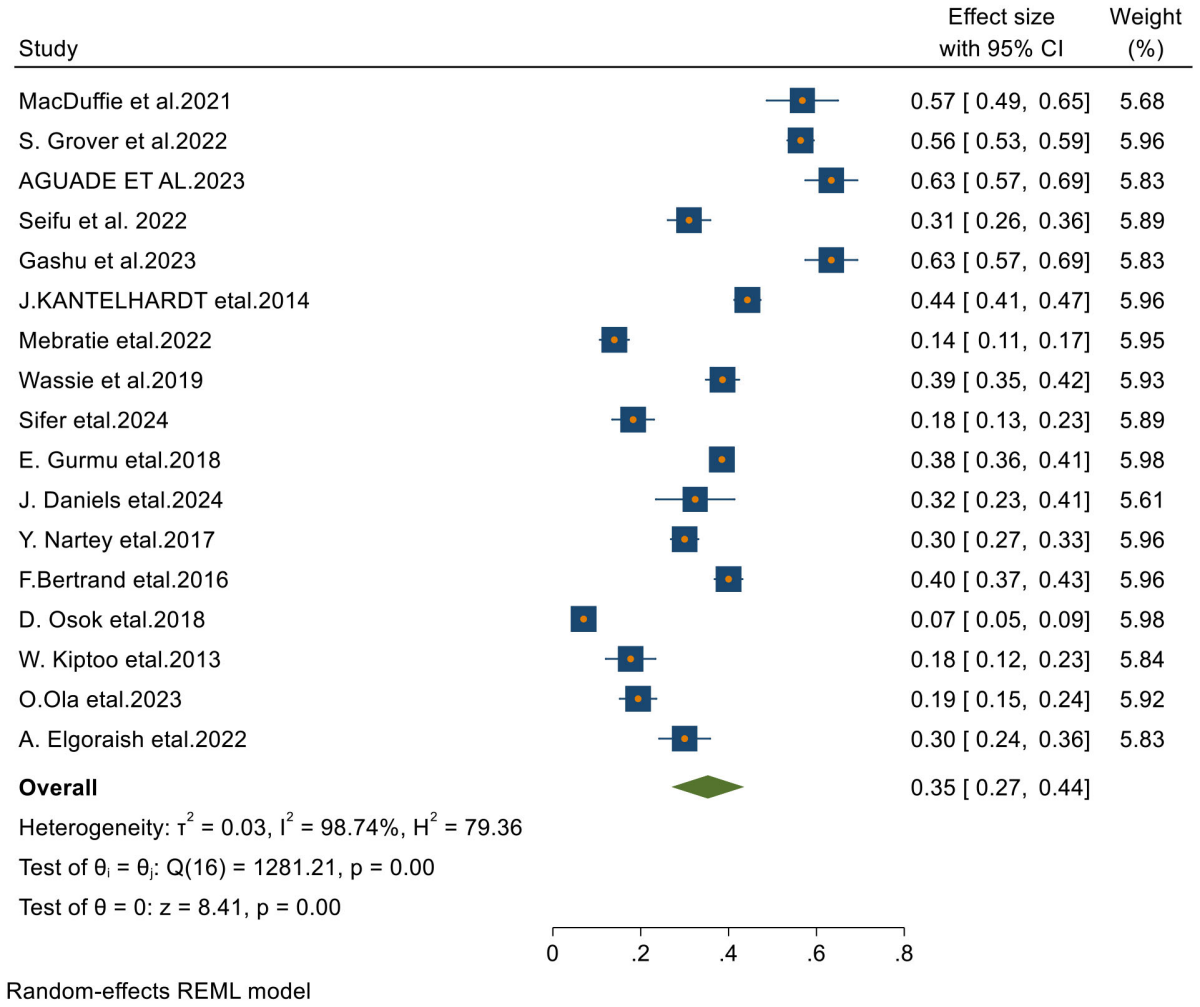
Forest plots of 5-year survival rates of patients with cervical cancer in Sub-Saharan countries.

Among the 23 articles included in this systematic review and meta-analysis, 17 studies reported the 5-year survival rates of patients with cervical cancer. Using a random-effects model, the overall pooled 5-year survival rate was found to be 35% (95% CI: 27–44), with an I² value of 98.74 and a p-value of less than 0.001 (**Figure 6**).

### Publication bias and heterogeneity

#### Publication bias

**Figure 7:**
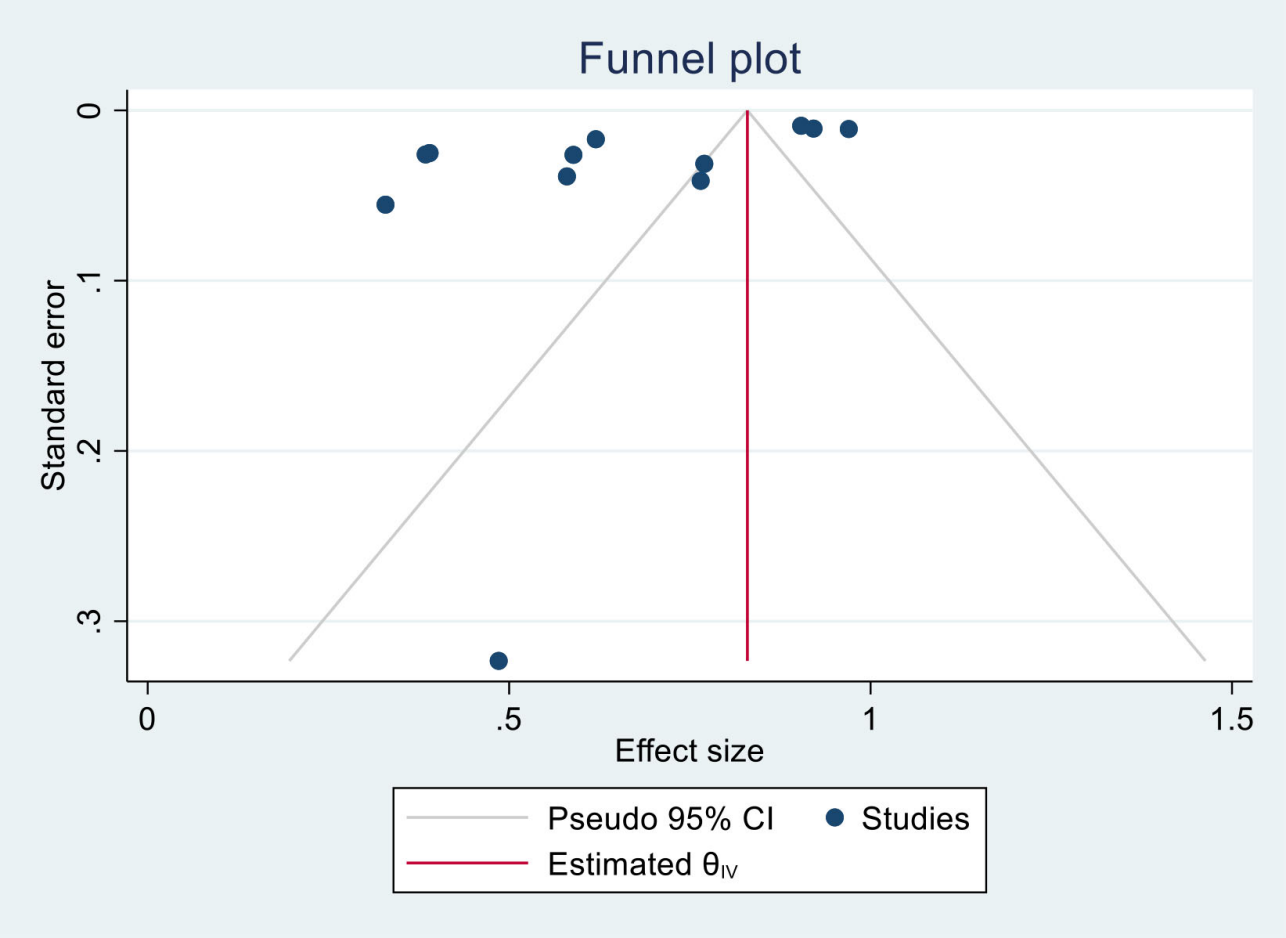
Funnel plots of 1-year survival rate of patients with cervical cancer in Sub-Saharan African countries.

**Figure 8:**
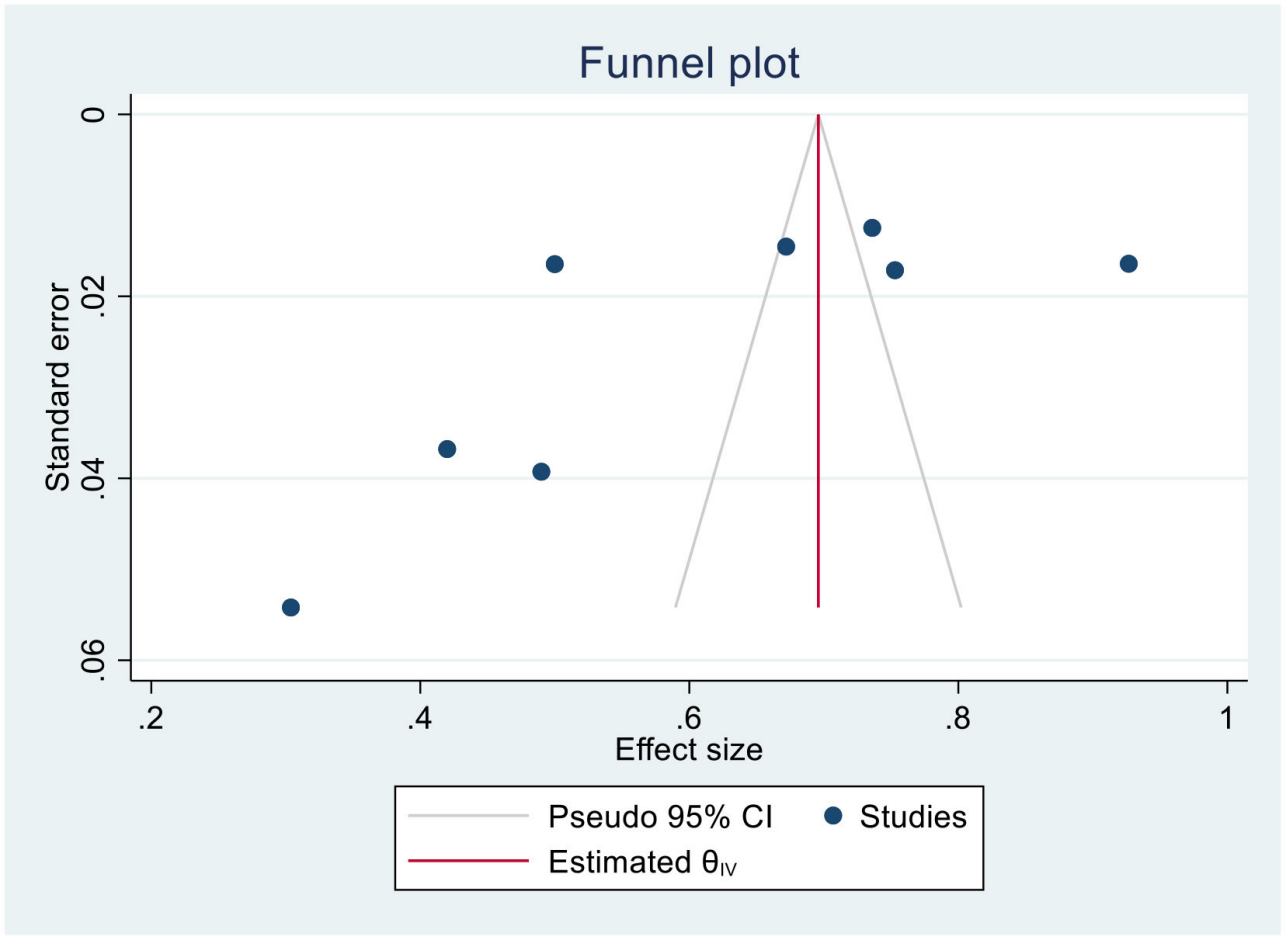
Funnel plots of 2-year survival rate of patients with cervical cancer.

**Figure 9:**
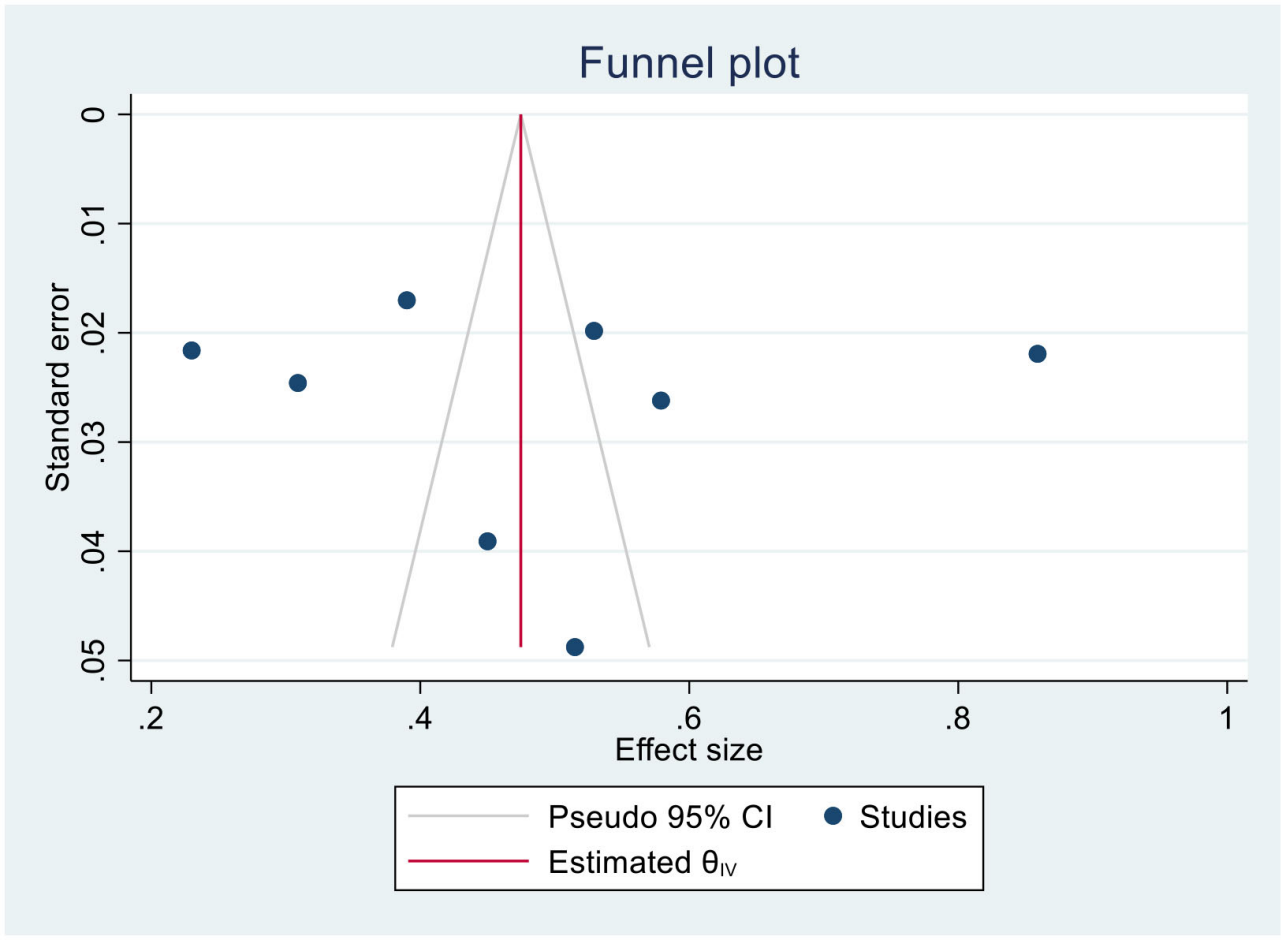
Funnel plots of 3-year survival rate of patients with cervical cancer.

**Figure 10:**
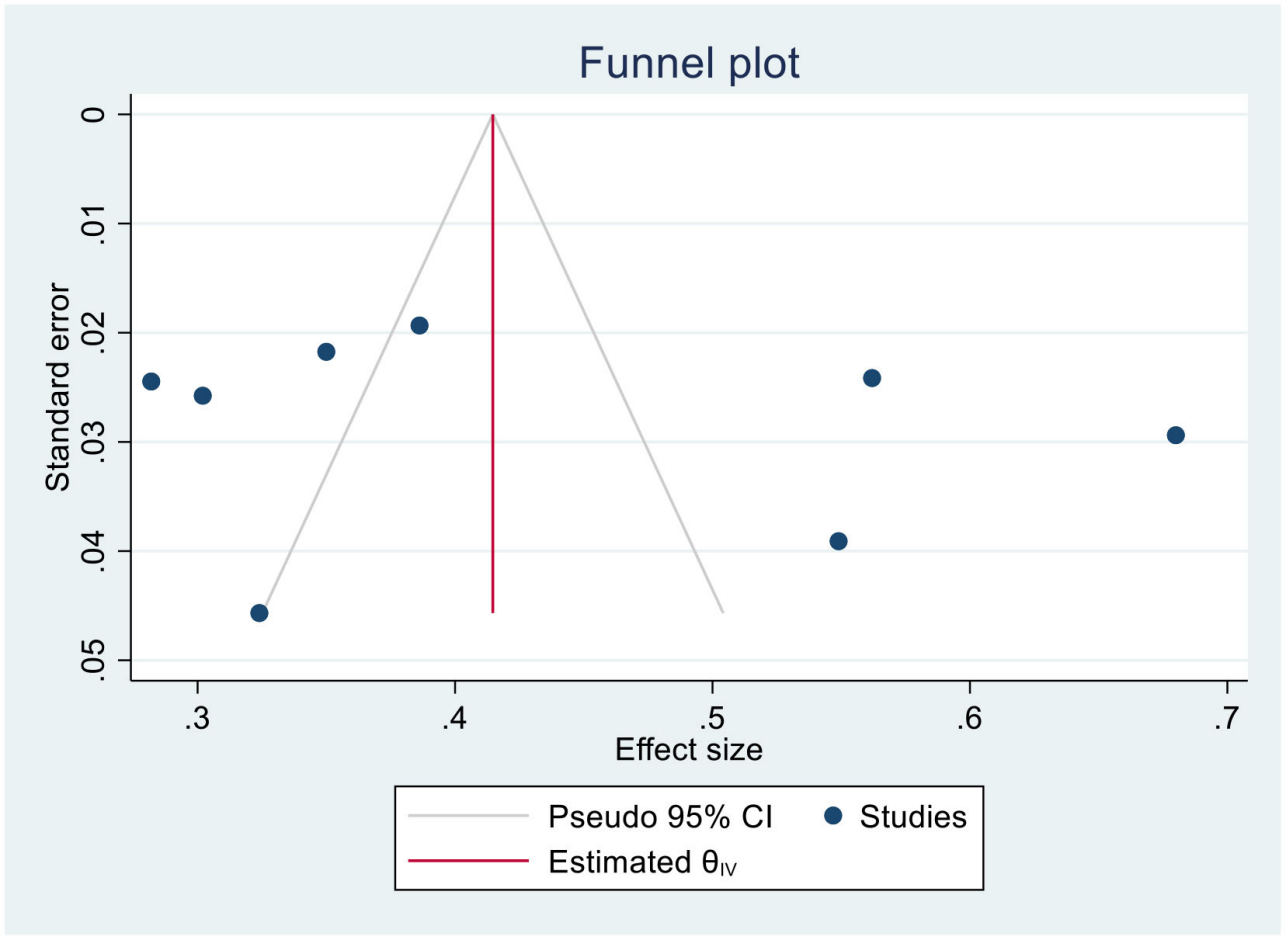
Funnel plots of 4-year survival rate of patients with cervical cancer.

**Figure 11:**
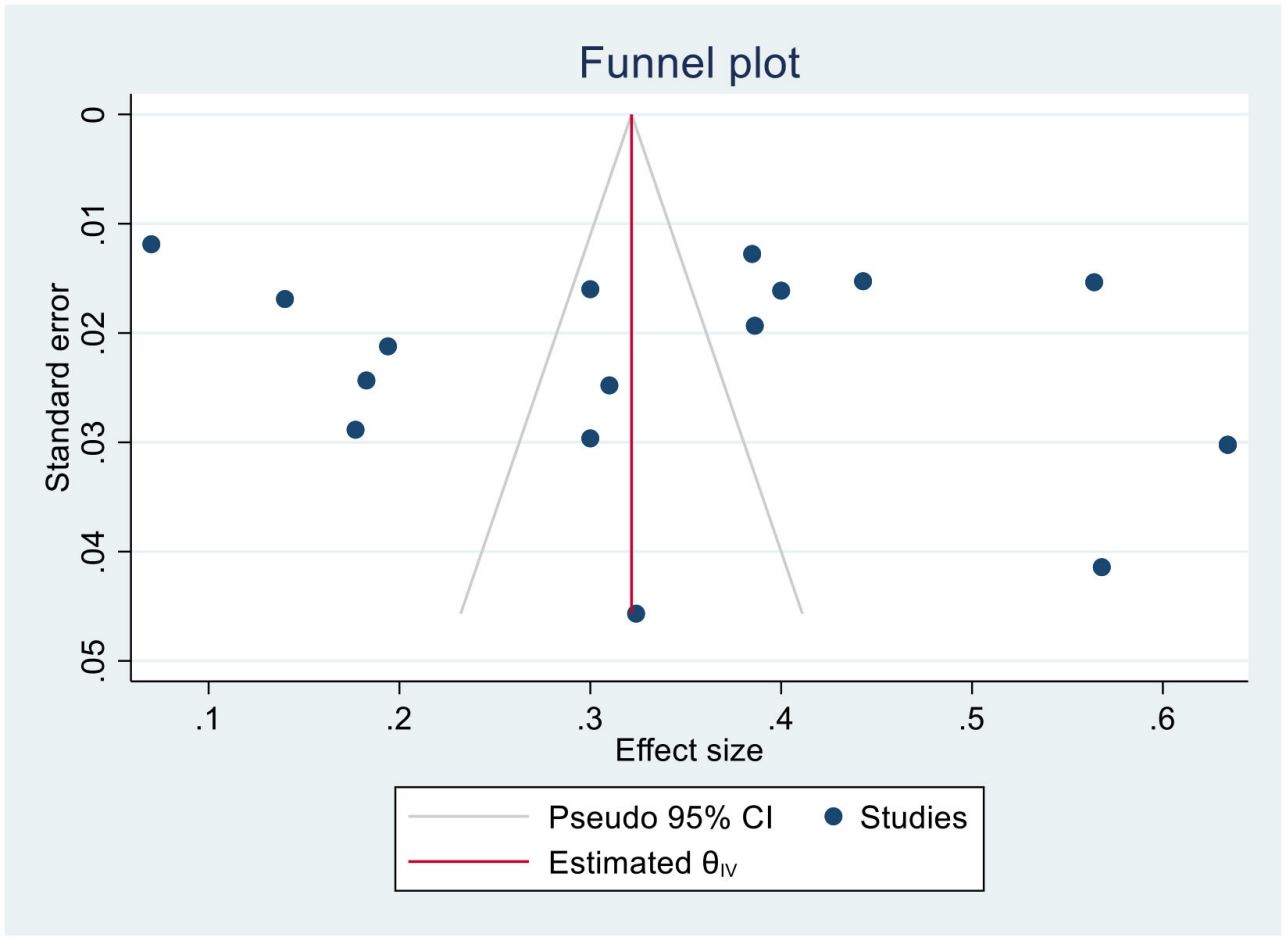
Funnel plots of 5-year survival rate of patients with cervical cancer.

We generated funnel plots to investigate the presence of publication bias in the cervical cancer survival rates at 1, 2, 3, 4, and 5 years in Sub-Saharan African countries. The results of the Egger test confirmed the presence of publication bias specifically for the 1-year and 2-year survival rates, as indicated by significant p-values shown below.

(One-year bias: −1.2453, 95% CI = −3.753 to 1.2620, P = 0.330) (**Figure 7**).

(Two- year bias: −10.804, 95% CI = −17.397 to − 4.873075, P = 0.001) (**Figure 8**).

(Three-year bias: 1, 95% CI = −0.8988 to 2.8988, P = 0. 302) (**Figure 9**).

(Four- year bias: 1.851, 95% CI = −11.125 to 14.828, P = 0.78) (**Figure 10**).

(Five- year bias: 5.04, 95% CI = −3.612 to 13.7053, P = 0.253) (**Figure 11**).

#### Trim-and-fill analysis for 1-year survival rate

**Figure 12:**
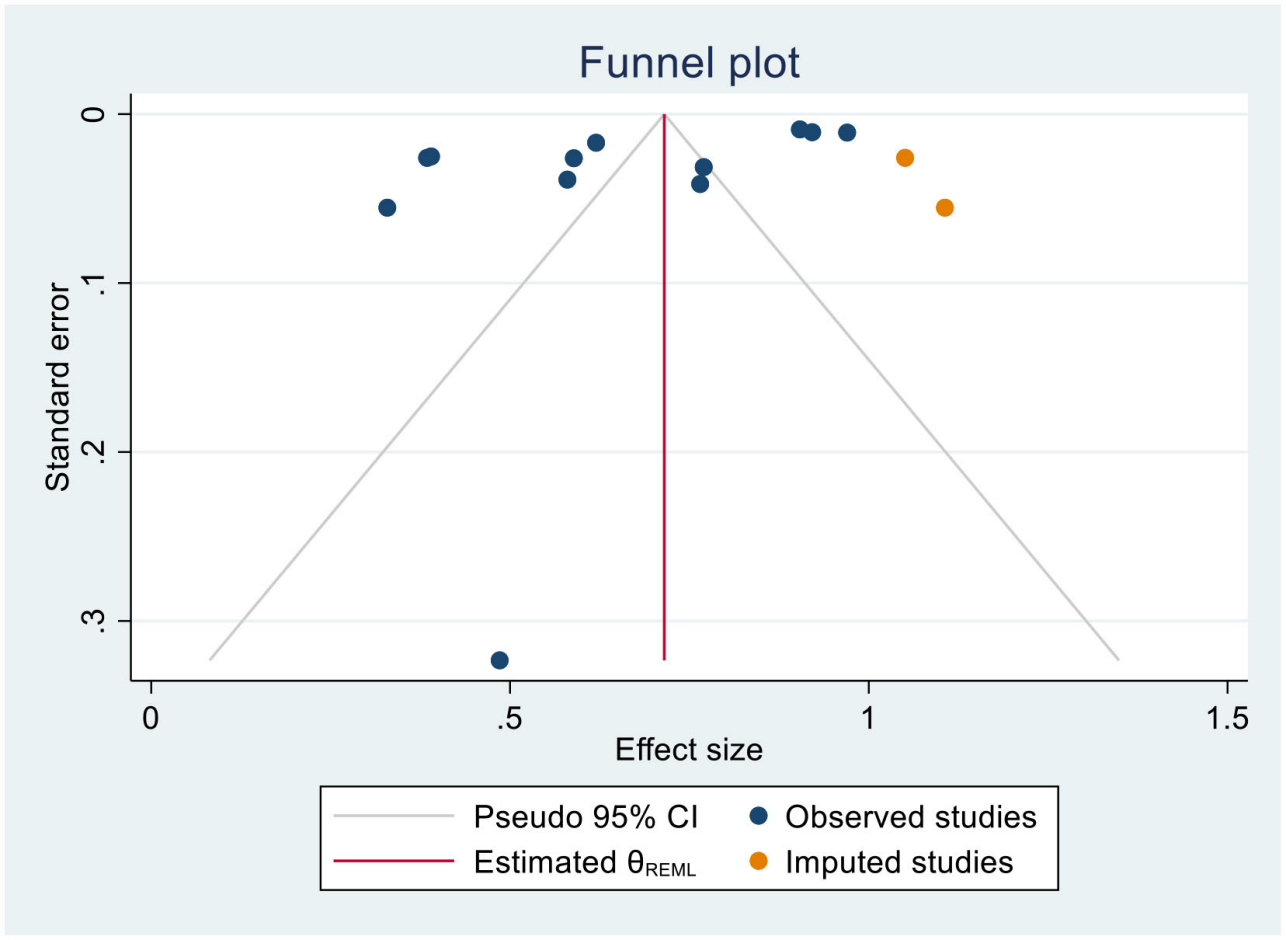
Trim-and-fill analysis filled funnel plot with 95% confidence limits of the pooled 1-year survival rate of patients with cervical cancer in Sub-Saharan African countries.

In this meta-analysis, due to the presence of publication bias in the 1-year survival rate of patients with cervical cancer, we executed a trim-and-fill analysis by using a random- effects model; the filled meta-analysis results showed that two studies were filled, which increases the number of studies from 12 to 14 with the pooled estimate of 1-year survival rate of patients with cervical cancer in Sub-Saharan African countries was 71.5 (95% CI, 57.7–85.3, p < 0.0001) (**Figure 12**). The pooled magnitude was changed from 72.0% (95% CI, 58.7–85.3) to 71.5% (95% CI 57.7–85.3) after trim- and- fill analysis. The egger test only detected minimal publication bias; however, because the later CI includes the first pooled size, there is no conspicuous difference between them which affects the final effect sizes.

#### Trim-and-fill analysis for 2- year survival status

In this meta-analysis, we found evidence of publication bias in the 2-year survival rates of patients with cervical cancer, as indicated by Egger’s test. To further evaluate the potential impact of this bias, we performed a trim-and-fill analysis using a random- effects model. The results indicated that no studies were identified as missing or imputed, suggesting that the original meta-analysis did not require adjustments for missing studies related to publication bias. Consequently, the pooled estimate of the 2-year survival rate remained unchanged following the trim-and-fill analysis. Although Egger’s test identified only a slight degree of publication bias, this minimal bias did not significantly affect the overall effect size or alter the conclusions of the meta-analysis. Therefore, the robustness of the pooled effect estimates was preserved, and the observed results can be deemed reliable despite the minor bias detected.

### Subgroup analysis

Due to the presence of heterogeneity within the included studies, a subgroup analysis based on country, sample size, and year of publication was conducted to identify the source of heterogeneity for each year of cervical cancer survival rates (1, 2, 3, 4, and 5 years).

#### Subgroup analysis of 1-year survival rate

The subgroup analysis of the 1-year survival rate for patients with cervical cancer revealed significant differences across countries. The lowest survival rate was observed in Nigeria (33.0%), whereas the highest was in Ethiopia (90%). When analyzed by year of publication, studies published before 2020 had a higher pooled survival rate (68%) compared to those published in 2020 and after (64%). Additionally, studies with larger sample sizes (≥500 participants) reported a higher survival rate (82%) compared to those with smaller sample sizes (<500 participants), which had a survival rate of 59% (**Table 2**).

**Table 2:** Subgroup analysis of 1- and 2 -year survival rates of patients with cervical cancer.

	Variables	Characteristics	Included studies	Number of study participants	Survival rate with 95% CI	I^2^, p-value
Subgroup analysis of 1-year survival rates	Countries	Ethiopia	4	2,377	90 [82–97]	97.71, < 0.001
		Ghana	2	926	69 [55–83]	90.4, < 0.001
		Kenya	2	517	59 [54–63]	0.01, < 0.001
		Nijeria	1	72	33 [22–44]	… …
		Côte d’Ivoire	1	353	38 [33–44]	……
		Rwanda	1	379	39 [34–44]	….
		Sudan	1	239	49 [15–112]	–
	Years of publication	Before 2020	5	2,941	68 [46–89]	99.60, < 0.001
		2020 and above	7	1,670	64 [46–81]	98.58, < 0.001
	Study participants	500 and greater	3	2,514	82 [62–101]	99.52, < 0.001
		Less than 500	9	2,097	59 [44–74]	98.43, < 0.001

	Variables	Characteristics	Included studies	Number of study participants	Survival rate with 95% CI	I^2^, p-value
Subgroup analysis of 2- year survival rates	Countries	Ethiopia	4	2,125	71 [51–92]	99.28, < 0.001
		Ghana	1	923	50 [47–53]	…., < 0.001
		Kenya	1	162	49 [41–57]	…, < 0.001
		Nijeria	1	72	30 [22–41]	… …
		Botswana	1	1,043	67 [64–70]	….
	Years of publication	Before 2020	4	2,688	58 [37–78]	99.24, < 0.001
		2020 and above	4	1,637	61 [41–85]	99.03, < 0.001
	Study participants	500 and greater	4	3,659	67 [55–78]	98.31, < 0.001
		Less than 500	4	666	54 [27–81]	98.52, < 0.001

#### Subgroup analysis of 2- year survival rate

The subgroup analysis of the 2-year survival rate for patients with cervical cancer showed significant variation by country, year of publication, and sample size. Nigeria had the lowest survival rate (30%), whereas Ethiopia had the highest (71%). Studies published in 2020 and after had a higher pooled survival rate (63%) compared to those published before 2020 (30%). Larger studies with ≥500 participants reported a higher survival rate (67%) compared to those with fewer participants (<500), which had a survival rate of 54% (**Table 2**).

#### Subgroup analysis of 3-year survival

The subgroup analysis of the 3-year survival rate for patients with cervical cancer revealed notable differences across countries, publication years, and sample sizes. The lowest survival rate was found in Rwanda (23%), whereas Ethiopia had the highest (69%). Studies published before 2020 had a higher pooled survival rate (50%) compared to those published in 2020 and after (47%). Interestingly, studies with smaller sample sizes (<500 participants) showed a slightly higher survival rate (49%) than those with larger sample sizes (≥500 participants), which had a survival rate of 46% (**Table 3**).

**Table 3:** Subgroup analysis of 3- and 4-year survival rates of patients with cervical cancer.

	Variables	Characteristics	Included studies	Number of study participants	Survival rate with 95% CI	I^2^, p-value	
Subgroup analysis of 3 years	Countries	Ethiopia	2	886	69 [37–102]	99.20, < 0.001
		Ghana	2	926	44 [32–57]	82.92, = 0.02
		Kenya	2	517	52 [39–64]	86.69, = 0.01
		Côte d’Ivoire	1	353	31 [26–36]	….
		Rwanda	1	379	23 [19–27]	….
	Years of publication	Before 2020		1,810	50 [39–61]	95.58, < 0.001
		2020 and above		1,251	47 [26–69]	98.72, < 0.001
	Study participants	500 and greater		1,455	46 [32–60]	96.48, < 0.001
		Less than 500		1,606	49 [31–67]	98.52, < 0.001
	Variables	Characteristics	Included studies	Number of study participants	Survival rate with 95% CI	I^2^, p-value	
Subgroup analysis of 4 years	Countries	Ethiopia	4	1,630	48 [32–65]	97.06, < 0.001	
		Ghana	1	105	32 [23–41]	….	
		Kenya	2	643	45 [25–64]	94.95, = 0.01	
		Nigeria	1	343	28 [23–33]	….	
	Years of publication	Before 2020	2	1,115	33 [37–40]	35.38, = 0.21	
		2020 and above	6	1,606	45 [32–59]	96.95, < 0.001	

#### Subgroup analysis of 4-year survival

The subgroup analysis of the 4-year survival rates for patients with cervical cancer highlighted significant differences based on country and publication year. The lowest survival rate was observed in Nigeria (28%), whereas Ethiopia had the highest rate (48%), with individual study variations ranging from 30.2% to 68%. In terms of publication year, studies published before 2020 had a lower pooled survival rate of 33%, whereas those published in 2020 and after showed a higher rate of 45% (**Table 3**). These findings underscore variability in survival outcomes across different contexts.

#### Subgroup analysis of 5- year survival rate

The subgroup analysis of 5-year survival rates for patients with cervical cancer indicated considerable variations. Kenya recorded the lowest survival rate at 12%, whereas Botswana reported the highest rate at 90%, with individual study rates ranging from 12% to 63%. When examining the year of publication, studies published prior to 2020 had a pooled survival rate of 31%, which was lower than the 38% reported in studies published in 2020 and thereafter. Additionally, studies with 500 or more participants demonstrated a higher pooled survival rate of 41%, in contrast to a lower rate of 32% in studies with fewer than 500 participants (**Table 4**).

**Table 4:** Subgroup analysis of 1-year survival rates of patients with cervical cancer.

Variables	Characteristics	Included studies	Number of study participants	Survival rate with 95% CI	I^2^, p-value
Countries	Botswana	2	1,186	56 [54–59]	0.04, 0.93
	Ethiopia	8	4,266	39 [26–51]	98.84, < 0.001
	Ghana	3	1,849	34 [28–41]	86.41, < 0.001
	Kenya	2	656	12 [2–22]	91, < 0.001
	Nijeria	1	343	19 [15–24]	… …
	Sudan	1	239	30 [24–36]	–
Years of publication	Before 2020	7		31 [21–41]	98.66, < 0.001
	2020 and above	10		38 [26–51]	98.44, < 0.001
Study participants	500 and greater	6	5,387	41 [34–48]	96.83, < 0.001
	Less than 500	11	3,152	32 [20–44]	98.64, < 0.001

### Sensitivity analysis of the 1-, 2-, 3-, 4-, and 5- year survival status of cervical cancer

The sensitivity analysis of the 1-, 2-, 3-, 4-, and 5-year survival rates for patients with cervical cancer in Sub-Saharan Africa was conducted to assess the robustness of the pooled survival estimates. Each analysis employed a “leave-one-out” approach to examine whether the results were influenced by any single study. Across all time frames, the findings indicated that the survival estimates were stable and not significantly impacted by the exclusion of individual studies, confirming the robustness of the pooled results.

#### Sensitivity analysis of the 1-year survival

We performed a leave-one-out sensitivity analysis to investigate potential sources of heterogeneity in the random pooled 1-year survival rates of patients with cervical cancer in Sub-Saharan countries. The results of this analysis suggested that our findings were robust and not significantly affected by any single study. The pooled estimated 1-year survival rate varied from 0.62 (95% CI: 0.49, 0.75) to 0.68 (95% CI: 0.55, 0.81) upon the exclusion of individual studies (**Table 5**).

**Table 5:** Sensitivity analysis of 4-year survival rates for each study being omitted with 95% CI.

Omitted study	One-year survival rate %	95% CI	p-value
J. Kantelhardt et al., 2014	0.63	[0.49, 76]	0.00
Wassie et al., 2019	0.63	[0.49, 0.76]	0.00
Teshome et al., 2024	0.64	[0.50, 0.78]	0.00
Sifer et al., 2024	0.62	[0.49, 0.75]	0.00
J. Daniels et al., 2024	0.64	[0.50, 0.78]	0.00
Y. Nartey et al., 2017	0.66	[0.51, 0.80]	0.00
E. Mwaliko et al., 2023	0.66	[0.52, 0.80]	0.00
O. Maranga et al., 2013	0.66	[0.52, 0.80]	0.00
Musa et al., 2016	0.68	[0.56, 0.81]	0.00
P. Boni et al., 2023	0.68	[0.55, 0.81]	0.00
J. DeBoer et al., 2022	0.68	[0.55, 0.81]	0.00
A. Elgoraish et al., 2022	0.66	[0.52, 79]	0.00

#### Sensitivity analysis of the 2- year survival

We conducted a leave-two-out sensitivity analysis to further explore the potential sources of heterogeneity in the random pooled 2- year survival rates among patients with cervical cancer in Sub-Saharan countries. This analysis indicated that our findings were robust and not influenced by any single study. The pooled estimated 1-year survival rate ranged from 0.56 (95% CI: 0.43, 0.68) to 0.56 (95% CI: 0.43, 0.68) following the removal of individual studies (**Table 6**).

**Table 6:** Sensitivity analysis of 4-year survival rates for each study being omitted with 95% CI.

Omitted study	Two- year survival rate %	95% CI	p-value
S. Grover et al., 2022	0.59	[0.43, 0.76]	0.00
J. Kantelhardt et al., 2014	0.58	[0.43, 0.74]	0.00
Wassie et al., 2019	0.58	[0.43, 0.74]	0.00
Teshome et al., 2024	0.63	[0.48, 0.78]	0.00
Sifer et al., 2024	0.56	[0.43, 0.68]	0.00
F. Bertrand et al., 2016	0.62	[0.46, 0.78]	0.00
E. Mwaliko et al., 2023	0.62	[0.46, 0.78]	0.00
Musa et al., 2016	0.64	[0.51, 0.78]	0.00

#### Sensitivity analysis of the 3-year survival

We conducted a leave-one-out sensitivity analysis to further investigate potential sources of heterogeneity in the random pooled 3-year survival rates of patients with cervical cancer in Sub-Saharan countries. This analysis demonstrated that our findings were robust and not significantly affected by any single study. The pooled estimated 3-year survival rate remained consistent, ranging from 0.56 (95% CI: 0.43, 0.68) to 0.56 (95% CI: 0.43, 0.68) with the exclusion of individual studies (**Table 7**).

**Table 7:** Sensitivity analysis of 4-year survival rates for each study being omitted with 95% CI.

Omitted study	Three- year survival rate %	95% CI	p-value
Wassie et al.2019	0.48	[0.32, 0.63]	0.00
Sifer et al., 2024	0.43	[0.33, 0.52	0.00
J. Daniels et al., 2024	0.48	[0.32, 0.63]	0.00
Y. Nartey et al., 2017	0.50	[0.34, 0.65]	0.00
E. Mwaliko et al., 2023	0.49	[0.33, 0.64]	0.00
O. Maranga et al., 2013	0.47	[0.32, 0.62]	0.00
P. Boni et al., 2023	0.51	[0.36, 0.65]	0.00
J. DeBoer et al., 2022	0.52	[0.39, 0.65]	0.00

#### Sensitivity analysis of the 4-year survival

We performed a leave-two-out sensitivity analysis to further investigate the potential sources of heterogeneity in the random pooled 4-year survival rates of patients with cervical cancer in Sub-Saharan countries. This analysis indicated that our findings were robust and not significantly impacted by any single study. The pooled estimated 4-year survival rate varied from 0.39 (95% CI: 0.31, 0.48) to 0.45 (95% CI: 0.34, 0.56) following the removal of individual studies (**Table 8**).

**Table 8:** Sensitivity analysis of 4-year survival rates for each study being omitted with 95% CI.

Omitted study	Four- year survival rate %	95% CI	p-value
Gashu et al., 2023	0.45	[0.34, 0.56]	0.00
Mebratie et al., 2022	0.41	[0.30, 0.52]	0.00
Wassie et al., 2019	0.44	[0.32, 0.55]	0.00
Sifer et al., 2024	0.39	[0.31, 0.48]	0.00
J. Daniels et al., 2024	0.44	[0.33, 0.56]	0.00
D. Osok et al., 2018	0.44	[0.33, 0.56]	0.00
E. Mwaliko et al., 2023	0.41	[0.30, 0.52]	0.00
O. Ola et al., 2023	0.45	[0.34, 0.56]	0.00

#### Sensitivity analysis of 5- year survival

We conducted a leave-two-out sensitivity analysis to further explore the potential sources of heterogeneity in the random pooled 5- year survival rates among patients with cervical cancer in sub-Saharan countries. This analysis indicated that our findings were robust and not influenced by any single study. The pooled estimated 5- year survival rate ranged from 0.0.26 (95% CI: 0.28, 0.45) to 0.37 (95% CI: 0.29, 0.45) following the removal of individual studies (**Table 9**).

**Table 9:** Sensitivity analysis of 4-year survival rates for each study being omitted with 95% CI.

Omitted study	Five- year survival rate %	95% CI	p-value
MacDuffie et al., 2021	0.34	[0.26, 0.42]	0.00
S. Grover et al., 2022	0.34	[0.26, 0.42]	0.00
Aguade et al., 2023	0.34	[0.26, 0.41]	0.00
Seifu et al., 2022	0.36	[0.27, 0.44]	0.00
Gashu et al., 2023	0.34	[0.26, 0.41]	0.00
J. Kantelhardt et al., 2014	0.35	[0.26, 0.43]	0.00
Mebratie et al., 2022	0.37	[0.28, 0.45]	0.00
Wassie et al., 2019	0.35	[0.26, 0.44]	0.00
Sifer et al., 2024	0.26	[0.28, 0.45]	0.00
E. Gurmu et al., 2018	0.35	[0.26, 0.44]	0.00
J. Daniels et al., 2024	0.35	[0.27, 0.44]	0.00
Y. Nartey et al., 2017	0.36	[0.27, 0.44]	0.00
F. Bertrand et al., 2016	0.35	[0.26, 0.44]	0.00
D. Osok et al., 2018	0.37	[0.29, 0.45]	0.00
W. Kiptoo et al., 2013	0.36	[0.28, 0.45]	0.00
O. Ola et al., 2023	0.36	[0.27, 0.44]	0.00
A. Elgoraish et al., 2022	0.36	[0.27, 0.44]	0.00

## Discussion

In Sub-Saharan Africa, the survival rates for patients with cervical cancer at 1, 2, 3, 4, and 5 years are alarmingly low compared to global averages. Several factors contribute to this situation, including late-stage diagnoses, limited access to comprehensive cancer care, and the absence of customized treatment strategies that address the individual needs of patients. The lack of personalized care, particularly regarding surgical interventions, has posed a significant challenge, resulting in suboptimal outcomes and increased recurrence rates. The discussion regarding the most effective surgical methods for treating cervical cancer adds complexity to treatment decisions in this region. Although radical hysterectomy, minimally invasive surgery (MIS), and other techniques have been examined, no definitive consensus exists on which approach strikes the best balance between reducing recurrence and enhancing survival (23). This uncertainty, coupled with insufficient infrastructure to deliver personalized care, significantly affects patients’ long-term outcomes (24, 25). Many patients experience recurrence or treatment-related complications, which could be mitigated with more personalized treatment plans. The study by Giacomo Corrado et al. compared recurrence patterns in FIGO stage IB1-IB2 cervical cancer between MIS and abdominal radical hysterectomy. It found no significant differences in recurrence patterns, disease-free survival, or overall survival between the two approaches, indicating that both are safe options for this stage. However, the study emphasizes the need for further research to identify risk factors contributing to recurrence, such as tumor characteristics and lymph node involvement.

Cervical cancer is a highly preventable disease, yet it causes over 300,000 deaths globally each year (7). This systematic review and meta-analysis assessed the survival rates of patients with cervical cancer in Sub-Saharan Africa at 1, 2, 3, 4, and 5 years after diagnosis. Our findings indicate that the 1-year survival rate was 65.0%, the 2-year survival rate was 60.0%, the 3-year rate was 48.0%, the 4-year survival rate was 42.9%, and the 5-year rate was 35.0%. The study highlights that survival rates have not shown improvement, particularly for the 4- and 5-year survival rates in recent years. Our results indicate that Kenya had the lowest 5-year survival rate at 7.0%, whereas Ethiopia had the highest at 63.3%. Despite Kenya having a more advanced healthcare infrastructure, better access to treatment, and broader screening programs, its lower 5-year survival rate compared to that of Ethiopia may be attributed to several factors. In Kenya, significant regional disparities, late-stage diagnoses despite screening efforts, and potential challenges related to treatment adherence and continuity of care could contribute to the lower survival rate. Furthermore, Kenya’s more comprehensive cancer registries may provide a more accurate and complete representation of outcomes, particularly in underserved areas. In contrast, Ethiopia’s higher survival rate might reflect data from specific hospitals or regions where early detection and continuous care are prioritized. There may also be underreporting of cases from rural or underserved areas that could skew the data. Additionally, differences in the burden of HIV co-morbidity, treatment adherence, and patient follow-up practices may help explain the survival disparities between the two countries. These findings suggest that survival rates are influenced not only by healthcare infrastructure but also by regional, social, and systemic factors. The lowest 1-year survival rate was observed in Nigeria at 32.9%, whereas the highest was found in Ethiopia at 96.99%.

As seen, the survival of cervical cancer in Sub-Saharan had much difference between 1-, 2-, 3-, 4-, and 5-year survival especially between 1 and 5 years. Perhaps the possible reasons of this might be, in many cases, cervical cancer is diagnosed at a more advanced stage in Sub-Saharan Africa due to limited access to screening and early detection programs. Earlier diagnosis generally leads to better survival because the cancer can be treated more effectively when it is detected early (26). Additionally, access to comprehensive cancer care, including surgery, radiotherapy, and chemotherapy, is often limited in Sub-Saharan Africa. This can lead to delays in receiving appropriate treatment, which negatively affects survival rates. Advanced treatments might not be available or accessible to all patients, influencing outcomes at different survival intervals (4). Of course, the up-to-date treatment methods should not be well addressed, and perhaps another reason for this observation is that Sub-Saharan Africa has high rates of HIV infection, which can compromise the immune system and make individuals more susceptible to infections and cancers like cervical cancer. HIV-positive women may have a higher risk of developing aggressive forms of cervical cancer and may have poorer survival outcomes (27). Overburdened healthcare systems, inadequate funding, and lack of prioritization of cancer care can lead to gaps in the delivery of essential services (13, 28). This affects the timely diagnosis, treatment, and follow-up care, influencing survival status over time of this cancer, and, finally, the survival at 1, 2, 3, 4, and 5 years is far apart to each other.

Compared to global data, cervical cancer survival rate in Sub-Saharan Africa is significantly lower. For instance, the 1-year survival rates of patients with cervical cancer in this study were 65.0%. This study was lower than a study done in China (29) with 1-year survival rate of cervical cancer of 96.3% and a study conducted in Malysia (30) with 1-year survival rate of patients with cervical cancer of 97.4%, and this study was also lower than a study done in Korea (31) with a 1-year survival rate of 93.2% and another study done in Korea (32) and a study done in The Netherlands (30) with 1-year survival rate of 87%. This disparity may be due to late stage of Diagnosis of cervical cancer in many Sub-Saharan African countries, as compared to countries like China, Malaysia, Korea, and The Netherlands. Late diagnosis is commonly due to a lack of routine screening programs, leading to poorer survival rates (7). Additionally, differences in healthcare infrastructure and access to medical care, including specialized cancer treatment facilities, could contribute to the lower survival rates, as high-income countries typically have better access to advanced diagnostic and treatment options, such as surgery, radiation therapy, and chemotherapy (33). The result of this study was in track with a study done in India (34), which reports that the 1-year survival rate of patients with cervical cancer was 67.3%.

In this study, the 2- year survival rate of patients with cervical cancer among Sub-Saharan women was 60%. This study was lower than a study done in Korea (31), with the 2-year survival rates of patients with cervical cancer in Korea of 86.8%, and another study done in Korea (32) with 2- year survival rate of patients with cervical cancer was 90.6%. This might be because, in most Sub-Saharan African countries, cervical cancer is often detected at more advanced stages due to limited access to regular screening programs, whereas, in Korea, early detection, which is more common due to widespread screening programs, is crucial for effective treatment and better survival outcomes (3). Additionally, as effective follow-up care is essential for managing patients with cervical cancer and managing complications, Sub-Saharan African countries’ challenges in healthcare delivery, such as lack of consistent follow-up care, may negatively impact long-term survival rates as compared with developed countries like Korea (1). However, this study was in line with a study done in Bhutan with 75.6% (35).

In regard to the 3-year survival rate of cervical cancer in Sub-Saharan Africa, this study reports that only 48% of patients with cervical cancer survived up to 3 years after diagnosis, which was significantly lower than reported in other parts of the world, such as China (36) with 74.3%, two in Korea (31, 32) with 83.0% and 93.5%, respectively, Malaysia (30) with 89.1%, The Netherlands (37) with 87.0%, and study done in rural India (34) with 68.0%. This discrepancy can largely be attributed to limited access to routine cervical cancer screening programs, such as Pap smears and HPV testing, in Sub-Saharan Africa (5). The absence of these essential early detection methods often leads to diagnoses at more advanced stages of the disease, which are more challenging to treat and have poorer prognoses. Additionally, a lack of awareness about cervical cancer symptoms further contributes to women presenting with advanced disease stages, where treatment options are less effective, resulting in lower survivals rates (38). However, the result of this study was in agreement with a study done in India (39), which reports that the 1-year survival rate of patients with cervical cancer was 40%.

The 4-year survival rate of patients with cervical cancer in Sub-Saharan Africa, as identified in this systematic review and meta-analysis, was 42.9%, significantly lower than the survival rate reported in other regions, such as Bhutan (35) (62.3%) and Korea (31, 32) (80.7% and 95.3% in two separate studies). The lower 4-year survival rate of patients with cervical cancer in Sub-Saharan Africa compared to that in Bhutan and Korea can be attributed to several factors. In Sub-Saharan Africa, limited access to healthcare services, including cancer screening, early detection, and advanced treatment options, often leads to later-stage diagnoses and poorer outcomes. In contrast, Bhutan and Korea likely benefit from more comprehensive healthcare infrastructure, including better access to screening, higher-quality treatment options, and more widespread HPV vaccination coverage (40). Additionally, socio-economic disparities, cultural beliefs, social stigma, and differences in study design and population characteristics further contribute to the variations in survival rates between these regions.

The 5-year survival rate of cervical cancer in Sub-Saharan Africa was found to be 35%, which is significantly lower compared to that in global studies. For instance, survival rates were reported as 71% in Amsterdam (41), 55.41% in Bhutan (35), 79.59% and 64.1% in two separate studies from China (29, 36), 67% in Estonia (42), 70.2% and 71.3% in two separate studies in Japan (43, 44), 79.2% and 80.6% in two studies from Korea (31, 32), 71.1% in Malaysia (30), 66.6% in Mexico (45), 69% in The Netherlands (37), and 48.1% and 60.5% in two studies from India (34, 39). The significantly lower 5-year survival rate of cervical cancer in Sub-Saharan Africa can be attributed to a combination of late diagnosis, inadequate healthcare infrastructure, socio-economic challenges, the burden of co-morbidities like HIV/AIDS, and limited access to advanced treatment and lack of neoadjuvant chemotherapies. In recent studies, the combination of neoadjuvant chemotherapy followed by radical surgery has been shown to improve outcomes in patients with locally advanced cervical cancer. Mereu et al. (2023) reported in their retrospective single-center study that this approach can achieve favorable survival rates in selected patients (46).

In contrast, countries with higher survival rates benefit from early detection, advanced treatments, strong healthcare systems, and supportive public health policies (25, 40). However, the survival rate in Sub-Saharan Africa aligns more closely with studies conducted in Saudi Arabia (32.1%) (28) and India (32.5%) (39). It is also higher than the survival rate reported by the Ocean Road Cancer Institute (47), which was 26%.

## Strengths and limitations of the study

A major strength of this study lies in its comprehensive approach, which synthesizes data from multiple studies across Sub-Saharan Africa to provide a more precise estimate of cervical cancer survival rates. However, several limitations should be acknowledged. The differences in sample sizes, inclusion of only English-language articles, and the quality of reporting could introduce bias. One of the key limitations of this systematic review and meta-analysis is the limited number of studies reporting on 2-, 3-, and 4-year survival rates. Out of the 23 studies included in the analysis, only 8 provided data on these intermediate survival intervals, which may affect the reliability and generalizability of the pooled estimates for these time points. The scarcity of data could potentially introduce bias, as the studies reporting these outcomes may differ from those that did not in terms of population characteristics, treatment protocols, or healthcare settings. This limitation may also reduce the statistical power of the analysis, leading to wider CIs and less precise estimates for 2-, 3-, and 4-year survival rates. Despite these limitations, the data available provide valuable insights into survival trends, although caution should be taken when interpreting these findings. Future research with more comprehensive reporting of intermediate survival outcomes is needed to strengthen the evidence base.

## Conclusion

This systematic review and meta-analysis revealed that cervical cancer survival rates in Sub-Saharan Africa, particularly the 1-, 2-, 3-, 4-, and 5-year survival rates, are significantly lower than global averages. The pooled data show that 5-year survival can be as low as 35%, a result of multiple interconnected factors, including delayed diagnosis, limited access to effective treatments, inadequate healthcare infrastructure, and the high burden of co-morbidities such as HIV. These findings emphasize the urgent need for targeted interventions to improve early detection through expanded screening programs and to enhance access to timely, effective treatment. Addressing socio-economic barriers and healthcare system limitations is essential for improving survival outcomes. Public health initiatives, such as increasing HPV vaccination coverage and raising awareness about cervical cancer, are also critical in reducing incidence and improving survival. In conclusion, the low survival rates reflect deep challenges in the management of cervical cancer in Sub-Saharan Africa, highlighting the need for coordinated action from governments, healthcare systems, and international organizations to strengthen cervical cancer care and reduce mortality. Future research should focus on strategies for early detection, timely treatment, and long-term outcome monitoring to track improvements over time.

## Data Availability

The data analyzed in this study is subject to the following licenses/restrictions: The data that support the findings of this study are available on request from the corresponding author Tadele Emagneneh. Requests to access these datasets should be directed to tadeleemagneneh@gmail.com.
