# Supplementary material for: Survival status of women with cervical cancer in Sub-Saharan Africa: a systematic review and meta-analysis, 2024
Source: Front Oncol. 2025 Jan 7;14:1491840. doi: 10.3389/fonc.2024.1491840 (PMC11746072; doi:10.3389/fonc.2024.1491840)
Supplement: Supplementary file 1 [file Table1.docx]

**Supplementary file 1: Literature search strategy for** **Survival Status of Women with Cervical Cancer in Sub-Saharan Africa: A Systematic Review and Meta-Analysis, 2024.**

| 1.((Cervical Cancer OR cervical Neoplasm OR cervical malignancy OR cervical tumor OR cervical lesions) and survival rate OR treatment outcome OR prognosis OR status and (All Fields)) OR (Cervical Cancer Survival Sub-Saharan Africa) OR (Survival Outcomes Cervical Cancer Sub-Saharan Countries) OR (Cervical Cancer Prognosis Sub-Saharan Africa) OR (Survival Rate of Cervical Cancer Patients Sub-Saharan Africa) OR) Cervical Cancer Treatment Outcomes Sub-Saharan Africa)) |
| --- |
| 2. ((Sub-Saharan African * OR Sub-Saharan African countries*OR developing countries (All Fields) OR all sub-Saharan Countries* OR (All Fields) OR(Angola) OR (Benin) or (Botswana) OR (Burkina Faso) OR (Burundi )(Cabo Verde) OR (Cameroon) OR (Central African Republic) OR (Chad OR Comoros) OR (Congo), (Democratic Republic of Congo) OR (Cote d'Ivoire) OR (Equatorial Guinea) OR (Eritrea) OR (Eswatini )(Formerly Known as Swaziland) OR (Ethiopia) OR (Gabon( Gambia, The) OR (Ghana) OR (Guinea-Bissau) OR (Kenya( Lesotho) OR) OR (Madagascar) OR (Malawi) OR(Mali)OR (Mauritania) OR (Mauritius) OR (Mozambique) OR (Namibia) OR (Niger) OR (Nigeria) OR (Rwanda) OR (Sao Tome and Principe) OR (Senegal) OR (Seychelles) OR (Sierra Leone) OR (Somalia) OR (South Africa) OR (South Sudan) OR Sudan)) |
| 3. (English) |
| 4. (Article OR Dissertation OR grey literatures) |
| 5. (#1 OR #2 OR #3 OR #4) AND #5) |
